# Aging partially restores the efficacy of malaria vector control in insecticide-resistant populations of *Anopheles gambiae s.l*. from Burkina Faso

**DOI:** 10.1186/1475-2875-11-24

**Published:** 2012-01-23

**Authors:** Christopher M Jones, Antoine Sanou, Wamdaogo M Guelbeogo, N'Fale Sagnon, Paul CD Johnson, Hilary Ranson

**Affiliations:** 1 Liverpool School of Tropical Medicine, Pembroke Place, Liverpool L3 5QA, UK; 2 Centre National de Recherche et de la Formation sur Paludisme, 01 BP2208 Ouagadougou, Burkina Faso; 3 Robertson Centre for Biostatistics, Boyd Orr Building, University of Glasgow, Glasgow G12 8QQ, UK

## Abstract

**Background:**

The operational impact of insecticide resistance on the effectiveness of long-lasting insecticide nets (LLINs) and indoor residual spraying (IRS) is poorly understood. One factor which may prolong the effectiveness of these tools in the field is the increase in insecticide susceptibility with mosquito age. In this study, LLINs and IRS were tested against young (three to five days) and old (17-19 days) pyrethroid resistant *Anopheles gambiae s.l*. from Burkina Faso.

**Methods:**

Blood-fed adult *Anopheles gambiae s.l*. were collected from south-west Burkina Faso and identified to species/form level. Cohorts of the F1 progeny of *An. gambiae s.s*. S-forms were exposed to deltamethrin (0.05%) at three to five or 17-19 days post-emergence and tested for the frequency of the resistance allele 1014F. Isofemale lines of the M, S- form of *An. gambiae s.s*. and *Anopheles arabiensis *were exposed in WHO cone tests to either a) LLINs deployed in households for two years or (b) bendiocarb sprayed walls.

**Results:**

Mortality rates in response to deltamethrin (0.05%) increased from levels indicative of strong resistance in three to five day old F1 mosquitoes, to near full susceptibility in the 17-19 day old cohort. On exposure to LLINs sampled from the field, the mortality rate in isofemale lines was higher in older mosquitoes than young (OR = 5.28, CI 95% = 2.81-9.92), although the mortality estimates were affected by the LLIN tested. In general, the LLINs sampled from the field performed poorly in WHO cone bioassays using either laboratory susceptible or field caught mosquito populations. Finally, there was a clear relationship between mortality and age on exposure to bendiocarb-sprayed walls, with older mosquitoes again proving more susceptible (OR = 3.39, CI 95% = 2.35-4.90).

**Conclusions:**

Age is a key factor determining the susceptibility of mosquitoes to insecticides, not only in laboratory studies, but in response to field-based vector control interventions. This has important implications for understanding the epidemiological impact of resistance. If mosquitoes old enough to transmit malaria are still being suppressed with available insecticides, is resistance potentially having less of an impact than often assumed? However, the poor performance of LLINs used in this study in Burkina Faso, is a cause for concern and requires urgent investigation.

## Background

Long-lasting insecticide nets (LLINs) and indoor residual spraying (IRS) have set a benchmark in malaria prevention. Significant reductions in malaria burden and transmission have been reported using LLINs and IRS alone or in combination [1-3]. In a bid to achieve the longer term goal of elimination, National Malaria Control Programmes (NMCP), donors and global health initiatives are fervently scaling up deployment of these interventions across Africa [4,5]. In Burkina Faso for example, the NMCP has procured approximately eight million LLINs to ensure widespread coverage in malaria endemic areas in 2010 [6].

It is against this backdrop that the emergence of insecticide resistance in the major African malaria vectors (*Anopheles gambiae s.s*., *Anopheles arabiensis *and *Anopheles funestus*) represents a serious concern. The arsenal of insecticides available for public health is limited to only four classes of chemistry (pyrethroids, organophosphates, carbamates and the organochlorine DDT) with no further additions on the market since the 1970s. Pyrethroids remain the only insecticide available for LLINs; resistance to this class has developed in the major vectors and is now firmly established throughout Africa [7-10]. Although carbamates and organophosphates offer a promising solution to counter pyrethroid resistant mosquitoes [11,12], pockets of resistance to these insecticides are emerging in West Africa [13,14]. The urgent need for alternative insecticides in disease control has been recognized for some time [15,16], however it is only lately that strides in addressing this issue have been made [17].

Insecticide resistance in *An. gambiae s.s*. is tightly linked to the ecology and agricultural practices of Burkina Faso. A large cotton belt extends from the west of the country towards the south and it is here that a resistance management strategy based on the rotation of pyrethroids, carbamates/organophosphates and organochlorines has been adopted since the 1990s to prevent extensive damage from cotton insect pests [14]. In recent years, the area of cultivation and volume of insecticide sprayed to protect cotton yields has increased dramatically and as a result, the levels of pyrethroid and DDT resistance in *An. gambiae s.s*. from this part of the country are much greater than in the central and eastern areas [18,19]. Resistance to a carbamate (bendiocarb) and an organophosphate (fenitrothion) has been detected from the same region [20]. These observed resistance patterns are closely related to the relative frequency and distribution of both the *An. gambiae s.s*. species complex and target-site mutations associated with insecticide resistance. The S-molecular form of *An. gambiae s.s*. predominates throughout the cotton belt regions of the south-west. The sodium channel mutation *L1014F *(otherwise known as knockdown resistance, *kdr*) and the acetylcholinesterase mutation *G119S *(*ace-1*), which reduce sensitivity to pyrethroids/DDT and organophosphates/carbamates respectively, are both found in S-form *An. gambiae s.s*. of the cotton belt region of Burkina Faso. Here, the *kdr *allele is approaching fixation in the S-form and, in Soumousso, is at approximately 0.6 in the M-form (N'Fale *et al. *unpublished data). In contrast, *An. arabiensis *from the cotton belt remains largely susceptible to pyrethroids.

In an area of insecticide resistance, it is anticipated that the number of mosquitoes surviving LLINs and/or IRS will rise and, as a result, the ability of the local vector population to transmit disease will increase. The relationship between insecticide resistance and vectorial capacity is, however, poorly understood. Relationships between insecticide resistance and insect fitness, longevity, behaviour and parasite interactions may increase or, indeed, decrease the vectorial capacity of mosquitoes (reviewed by Rivero *et al. *[21]). One recently revisited aspect of this relationship is the decline in insecticide resistance observed with increasing mosquito age. Mosquitoes, which would otherwise be defined as 'resistant' according to the standard WHO susceptibility tests, gradually lose their tolerance to insecticides as they become older and this has been demonstrated in multiple species [22-27]. The very success of LLINs and IRS is based on their ability to reduce the daily survival rate of the *Anopheles *vector and prevent the completion of parasite development into the infectious sporozoite stage. If resistance declines in older mosquitoes, it may be expected that those insects which have lived long enough to become infectious, on entering a house, have a far greater chance of being killed on encountering either an LLIN or a sprayed wall. In this scenario, the intervention would continue to provide a continued degree of efficacy even in the presence of multiple insecticide resistance mechanisms. The reduced phenotypic resistance in older mosquitoes has been tested in several laboratory studies [25-27], but very few studies have explored this trait in natural populations. Furthermore, the impact of this on the effectiveness of vector control tools under field conditions is largely unexplored. The objective of this study was to determine whether insecticide-based malaria control activities remain effective against both young and old *An. gambiae s.s*. collected from an area of strong resistance in south-west Burkina Faso.

## Methods

### Study site and mosquito collections

Blood-fed female *Anopheles gambiae *were collected between 0700 and 0900 h from the inside of houses from the village of Soumousso, located in the south-west of Burkina Faso (N 11° 00' 46", W 04° 03' 25"). A total of six collections were undertaken during August to November 2010. Soumousso lies within the heart of a large cotton-growing region where crops such as maize and rice are also grown. For control of agricultural pests, insecticides are sprayed up to six times per year between June and October and rotated among pyrethroids, organophosphates and carbamates. Routine insecticide resistance monitoring has been performed in this area since 2008 as part of a WHO/TDR multi-country project and resistance to all major insecticide classes available for malaria control has been reported [20]. No centrally organized malaria control is conducted in this region and insecticide treated net usage is low. The pre-dominant *An. gambiae s.s*. sub-species found in Soumousso during the early part of the malaria transmission season is *An. gambiae s.s*. S-molecular form, whereas the number of *An. gambiae s.s*. M-form and *An. arabiensis *increases towards the end of the rainy season [20].

### Mosquito rearing and species identification

All mosquitoes were reared in the insectaries at the Centre National de Recherche et de Formation sur le Paludisme (CNRFP), Ouagadougou. Blood-fed female *An. gambiae *were placed individually in small cups for oviposition over a two to four-day period. Immediately after egg-lay, a single leg was removed from each F0 female parent and used directly for species identification following SINE-PCR which discriminates between S-form, M-form and *An. arabiensis *[28]. Mosquitoes which failed to lay eggs or could not be identified to species were removed from the study. Eggs and newly hatched larvae from each individual parent were then either pooled according to species status or raised separately as isofemale lines. Adult mosquitoes were provided with a sugar solution for feeding. Blood meals were provided to mosquitoes older than five days. Mosquitoes that constituted the older cohort (17-19 days old) used in the study had received two blood meals and completed at least two gonotrophic cycles.

### Deltamethrin susceptibility tests

In order to demonstrate an age-effect in the progeny of field caught mosquitoes, a minimum of 100 three to five-day and 17-19-day old female mosquitoes from each of two pooled S-form populations were exposed to WHO 0.05% deltamethrin impregnated papers for one hour. Only *An. gambiae s.s*. S-forms were used as this was the predominant species during the study period. The number of mosquitoes 'knocked down' was recorded at the end of exposure and mortality scored 24 h after. Control assays were performed throughout the experiment with a minimum of 25 mosquitoes exposed to non-insecticide treated papers.

### L1014F TaqMan assay

Genomic DNA (gDNA) was extracted from 12 *An. gambiae s.s*. S-form mosquitoes either exposed to deltamethrin (0.05%) or from the control assays (non-exposed). One percent of the total gDNA was used in a TaqMan allelic discrimination PCR described by Bass *et al. *[29] to detect the presence of the *1014F kdr *allele

### Long-lasting insecticide net assays

Three Permanet2.0^® ^LLINs were sampled from the village of Laye situated 35 km north-west of the capital Ouagadougou (N 12° 31' 35", W 1° 46' 21"). The nets had been deployed in the village for approximately 2 years prior to the study as part of the Burkina Faso NMCP and were exchanged for new LLINs with the householder upon retrieval. Cohorts of three to five- and 17-19-day old mosquitoes from individual isofemale lines were exposed to each LLIN in WHO cone assays. Batches of five mosquitoes from the two age-cohorts belonging to the same isofemale line were exposed to the same piece of LLIN for 3 minutes according to WHO guidelines [30]. Only isofemale lines, which produced more than 20 female adults were selected for testing to ensure sufficient numbers were available for bioassays on each age cohort. Knockdown was recorded 1 hour following exposure and mortality scored 24 h later.

### LLIN evaluation

The LLINs were transported to the Liverpool School of Tropical Medicine (LSTM) to assess the insecticidal properties of the nets. A minimum of 24 females from a laboratory susceptible *An. gambiae *strain (Kisumu) were exposed for 3 minutes in WHO cone assays against each LLIN and mortality recorded 24 h later.

An estimate of the concentration of deltamethrin on each net was performed using HPLC. For each net, five random samples (30 mm in diameter) were cut from different areas of the net (total area of 35.5 cm^2^). The five pieces were cut into smaller pieces into a 50 ml Falcon tube, 40 μl of DDT 2 mg/m^2 ^was added as an internal standard (normalise extraction), and acetone extracted by vortexing 3 × with 5 ml acetone, pooled and evaporated to dryness under a stream of nitrogen. The insecticide residue was recovered in 1 ml acetonitrile and filtered with a 17 mm PTFE 0.2 μm syringe filter (Chromacol LTD, UK) before analysis. HPLC analysis was performed by injection of 20 μl aliquots onto a reverse-phase Dionex Acclaim C18 column (120Ǻ, 250 × 4.6 mm, 5 μ, Dionex, Camberley, UK). A mobile phase of methanol/water 90:10 was used at a flow rate of 1 ml/min^-1^.

The quantity of deltamethrin remaining on the net (mg/m^2^) was calculated from the standard curve established with known concentrations of an authenticated deltamethrin peak detected using an Ultimate 3000 UV detector and analysed with Dionex Chromeleon software.

### Bendiocarb IRS assays

Three houses from the village of Laye were sprayed with bendiocarb (200 mg/m^2^) on 27 August, 2010 using Hudson sprayers. Two houses were composed of mud walls and one built of cement. Between three and 8 weeks later, approximately 10 mosquitoes from each age cohort from each isofemale line described above were exposed to the bendiocarb sprayed wall for 3 min using WHO cone tests. A maximum of five mosquitoes were used per cone (two cones) and knockdown and mortality were recorded as described above. The same area of each wall was used to test the different age groups from the same isofemale line. Mosquitoes from both age groups were exposed to a non-sprayed wall in a different house as a control throughout all bioassays.

The above 3 min exposure experiment was designed to maximise the chances of observing age related changes in mortality. However, as the WHO protocol for evaluating IRS involves a 30 min exposure, we repeated the experiment in May 2011. Three houses were sprayed as described above and mosquitoes collected from Soumousso were used for the bioassays. The only difference in methodology was that mosquitoes were exposed to the surfaces for 30 min, as opposed to 3 min.

### Statistical analysis

The exact 95% confidence intervals for mortality in bioassays were calculated according to the Binomial distribution using R version 2.12.2 for Windows [31]. A Fisher exact test (*p *< 0.05) was used to assess the statistical significance of differences in mortality between age groups.

Mortality from the LLIN and IRS isofemale experiments was modelled using binomial generalized linear mixed models (GLMM) with a logit link function in the R package "lme4" [32]. A separate model was derived for each experiment with the aim of estimating whether age had an effect on mosquito mortality (binary response variable) upon exposure to either a LLIN or a wall sprayed with bendiocarb. Mortality was primarily modelled as a function of three fixed factors; 'Age' (young (three to five days) or old (17-19 days)), 'Species' (*An. arabiensis, An. gambiae s.s*. S-form or *An. gambiae s.s*. M-form) and 'Treatment' (LLIN or sprayed wall labelled T1-T3). Furthermore, in both models, 'Family' was included as a random effect to allow the log odds of mortality to vary between families. Testing for interactions between categorical explanatory variables is problematic when there are zero frequencies within the contingency table (i.e. zero mortality within a subgroup). This results in parameter estimates whose likelihoods increase towards ± infinity so that maximum likelihood estimates do not exist [33]. For this reason, and to test for evidence of possible interactions, a small weighted constant (total = 0.5) was added to the mortality rates contributing to the zero cell count (no mortality) in the younger age-group exposed to LLIN#1 [33]. Akaike's Information Criterion (AIC) was used to provide a strength of evidence for each model tested [34]. The estimated odds ratio with 95% confidence intervals was calculated relevant to the control treatment. More detail on the modelling approaches can be found in Additional file 1. All GLMM analyses were performed in R version 2.12.2 for Windows [31]. The R-script is available from the author on request.

## Results

### Mosquito collections from Soumousso

**Figure 1:**
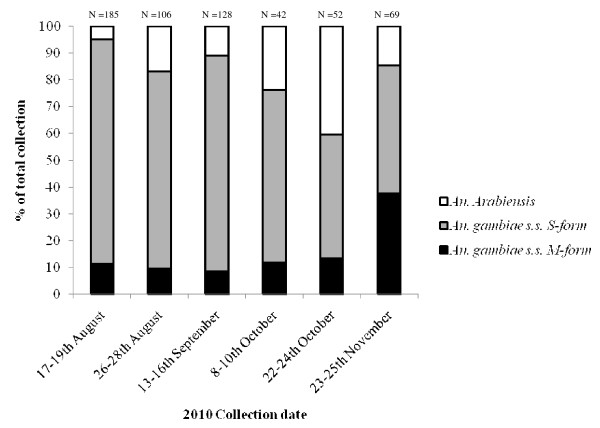
**The relative percentage of *Anopheles gambiae s.l*. caught during 2010 from Soumousso in south-west Burkina Faso**. F0 female *An. gambiae s.s*. caught from Soumousso were identified from each collection as *An. gambiae s.s*. M/S molecular form or *An. arabiensis *using SINE PCR [26]. The number identified from each collection is stated above the bar.

A total of 582 blood-fed *Anopheles gambiae s.l*. were collected from Soumousso and identified to species. *An. gambiae s.s*. S-form pre-dominated throughout all collections. Between August and September over 73% of all *An. gambiae s.s*. identified were *An. gambiae s.s*. S-form. The proportion of *An. arabiensis *and *An. gambiae s.s*. M-form was higher in October and November (Figure 1).

### Deltamethrin susceptibility tests

**Figure 2:**
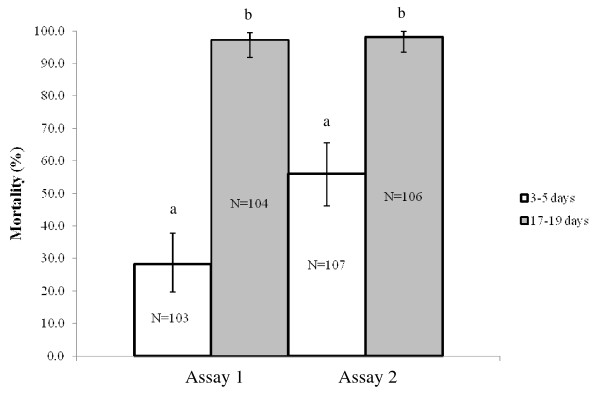
**The percentage mortality of *Anopheles gambiae s.s*. S-form from Soumousso following one-hour exposure to deltamethrin (0.05%) at three to five and 17-19 days old.** Two separate assays (Assay 1 and Assay 2) were performed on pooled S-form *An. gambiae s.s*. isofemale families. Different notations represent significant differences in mortality between age-groups within each test (*p *< 0.0001).

Pools of *An. gambiae s.s*. S-form from approximately 50 families were exposed to deltamethrin (0.05%) in WHO susceptibility tests. This was repeated on two separate occasions (Assay 1 and Assay 2). In each experiment two age cohorts were exposed. The percentage mortality in three to five-day old mosquitoes ranged from 28.2% (N = 103, 19.7-37.9%) to 56.1% (N = 107, 46.2-65.7%). Mortality significantly increased in the 17-19-day old mosquitoes for both experiments (*p *< 0.0001) reaching 97.1% (N = 104, 91.8-99.4%) and 98.1% (N = 106, 93.4-99.8%) (Figure 2). The percentage knockdown after 60 min exposure was high throughout all testing (between 92.4 to 100%) and did not differ between the age-cohorts. No control mortality was observed throughout the experiment. This experiment on pooled mosquitoes was performed to confirm that the age dependent resistance we had observed in earlier laboratory studies [25] was reproduced on field populations. The numbers of M-form and *An. arabiensis *were too low to repeat this experiment using these species/forms.

A sub-sample of 17-19-day old S-form *An. gambiae s.s*. was analysed for the *1014F kdr *allele from individuals dead following deltamethrin exposure and from non-exposed mosquitoes from the control bioassay. The frequency of *1014F *was 95.8% in both the test and control samples (N = 12 for each test). The high frequency of *1014F*, supplemented by recent data collected from Soumousso as part of the WHO/TDR project, suggests that *1014F *is at a very high frequency in this population and therefore variation in *1014F *frequency is unlikely to contribute to the differences in mortality seen here between age-groups.

### Long-lasting insecticide net assays

A total of 28 isofemale *An. gambiae s.s*. families from Soumousso (12 S-form, 11 M-form and five *An. arabiensis*) were exposed to LLINs at three to five days and 17-19 days old. The total number of mosquitoes exposed to each net and the number dead per isofemale are presented in Table 1.

**Table 1:** Mortality within *Anopheles gambiae s.l*. isofemale lines exposed to LLINs collected from Laye village

			three to five days			17-19 days		
**Net**	**Family No**.	Species*	**N**	**No. Dead**	**% Mortality**	**N**	**No. Dead**	**% Mortality**

LLIN#1	1	S	10	0	0.0	10	0	0.0

	7	S	10	0	0.0	11	4	36.4

	15	*An. arabiensis*	9	0	0.0	9	3	33.3

	39	M	11	0	0.0	10	0	0.0

	44	M	10	0	0.0	11	1	9.1

	104	S	10	0	0.0	10	0	0.0

	149	*An. arabiensis*	10	0	0.0	9	5	55.6

	208	M	10	0	0.0	10	0	0.0

	225	M	10	0	0.0	10	3	30.0

	250	M	10	0	0.0	9	4	44.4

	263	M	10	0	0.0	10	5	50.0

	279	M	10	0	0.0	13	5	38.5

LLIN#2	803	M	11	0	0.0	11	4	36.4

	815	*An. arabiensis*	11	4	36.4	11	11	100.0

	843	M	9	5	55.6	10	5	50.0

	858	M	10	3	30.0	10	1	10.0

	875	*An. arabiensis*	10	8	80.0	13	13	100.0

	883	*An. arabiensis*	8	7	87.5	15	13	86.7

	1062	S	9	1	11.1	15	0	0.0

LLIN#3	1156	S	11	2	18.2	7	0	0.0

	1157	S	9	0	0.0	7	0	0.0

	1165	S	9	1	11.1	7	1	14.3

	1196	S	11	0	0.0	5	0	0.0

	1131	S	10	0	0.0	10	1	10.0

	1135	S	9	0	0.0	8	0	0.0

	1188	S	11	0	0.0	10	0	0.0

	1198	M	10	0	0.0	9	1	11.1

	1203	S	9	0	0.0	10	1	10.0

*M = *An. gambiae s.s*. M-form; S = *An. gambiae s.s*. S-form

**Figure 3:**
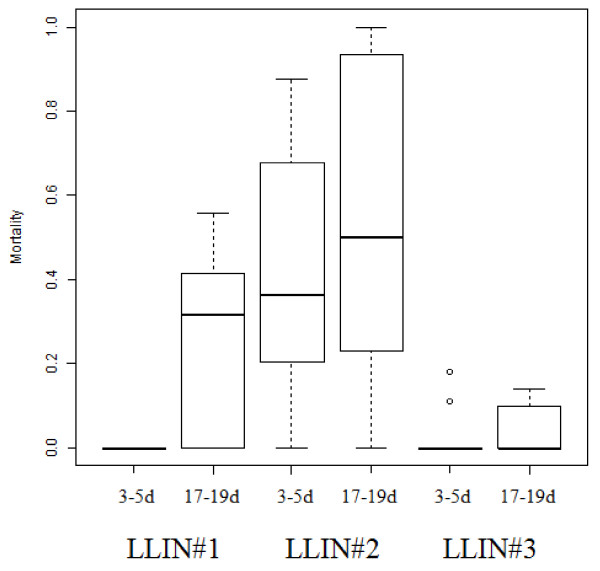
**The mortality of isofemale *Anopheles gambiae s.l*. from Soumousso in response to LLINs from the field**. The number of isofemale lines exposed to each net; LLIN#1 = 12, LLIN#2 = 7, LLIN#3 = 9.

The mortality of the isofemale *An. gambiae s.s*. from Soumousso varied between each of the LLINs used in the experiment and on the whole, the performance of the nets was poor. A boxplot showing the range of mortality within isofemale lines for each age cohort exposed to LLINs is given in Figure 3. All 12 of the isofemale lines tested on LLIN#1, and seven out of nine on LLIN#3, gave zero mortality when tested against the younger cohort (Figure 3). Against 17-19-day old mosquitoes, the performance was slightly better, with mortality ranging from 0-55.5% for LLIN#1 and 0-14.2% for LLIN#3. LLIN#2 performed better,, but even with this net, mortality rates ranged from 87.5% at best, to 0% at worst, against three to five-day old mosquitoes. The performance of each LLIN from the field was reflected in the mortality rates of the laboratory susceptible colony (Table 2). The concentration of deltamethrin on each of the nets varied between 1.0-2.0 mg/m^2 ^and is considerably lower than the original concentration on the net (55 mg/m^2^).

**Table 2:** The percentage mortality of the laboratory susceptible *Anopheles gambiae s.s*. Kisumu strain exposed to LLINs from Laye for three minutes in a WHO cone bioassay, and the concentration of deltamethrin remaining on each net

	WHO Cone Bioassay				Deltamethrin concentration*
**LLIN**	**N**	**Dead**	**Mortality (%)**	**CI 95%**	**mg/m^2^**

LLIN#1	36	19	52.8	35.5-69.6	1.0

LLIN#2	29	26	89.7	72.7-98.7	2.0

LLIN#3	24	6	25.0	9.8-46.7	1.9

*determined using HPLC

The relationship between age and resistance was not expected to differ between members of the *An. gambiae *complex and hence isofemale lines were not assigned to individual nets according to species. However, post hoc analysis did not indicate any evidence of a confounding effect of species (see Additional File 1 for more information).

Mosquito mortality was modelled as a function of three factors; 'Age', 'Species' and 'Treatment'. The maximal model incorporating all factors, and including 'Family' as a random effect, is given in Table 3. There was evidence for an interaction between 'Age' and 'Treatment' (ΔAIC = 13.2; Additional file 1), however, mortality estimates are artificially inflated due to the addition of a small weighted constant in the zero cell group (younger mosquitoes exposed to LLIN#1). Hence, to test for the affect of age, the GLMM omitting this interaction term is presented. A larger sample size would be required to improve estimates of the interaction between age and the LLIN tested. The criteria for selection of the fitted model (ΔAIC), estimated parameters, *z *values and *p *values are given in the Additional Information (Additional file 1).

**Table 3:** The estimated effect of mosquito age, *Anopheles gambiae s.l*. species and treatment on mortality in isofemale lines in response to either LLINs or IRS

		OR*	95% CI^#^	*P *value			OR	95% CI	*P *value
LLIN^aλ^					IRS^b^				

Age					Age				

	Young (3-5d)	1.00				Young (3-5d)	1.00		

	Old (17-19d)	5.28	2.81-9.92	< 0.0001		Old (17-19d)	3.39	2.35-4.90	< 0.0001

Species					Species				

	*An. arabiensis*	1.00				*An. arabiensis*	1.00		

	*An. gambiae *S-form	0.04	0.01-0.19	< 0.0001		*An. gambiae *S-form	1.56	0.97-2.51	0.065

	*An. gambiae *M-form	0.13	0.04-0.43	0.0007		*An. gambiae *M-form	0.93	0.41-2.10	0.855

Treatment									

	LLIN#1	1.00							

	LLIN#2	5.58	2.97-10.5	0.001					

	LLIN#3	1.06	0.56-2.00	0.987					

*IRS *= indoor residual spraying; *LLIN *= long-lasting insecticide net

*OR = odds ratio

^#^*CI *= confidence interval

^λ^Small weighted constant added to cells contributing to the zero cell count in the younger age-group for LLIN#1

a = A total of 28 isofemale lines were tested against three LLINs

b = A total of 30 isofemale lines were exposed to three walls sprayed with bendiocarb (0.1%)

According to the model, there was strong evidence that mosquito age had an effect on mortality. The older age group (17-19 days) were more likely to be killed on encountering the LLINs used in this experiment, compared with the younger cohort (three to five days) (Estimated OR = 5.28, 95% CI 2.81-9.92, p < 0.0001). It should be noted that this is a pooled estimate that assumes that the odds ratio is the same for each LLIN and species.

### Bendiocarb IRS assays

To determine the susceptibility of *An. gambiae s.s*. to bendiocarb sprayed on the walls in Laye, three isofemale lines from Soumousso (*An. gambiae s.s*. M-form) were exposed in WHO cone tests for 30 min. All mosquitoes were killed, suggesting complete susceptibility to bendiocarb from this area and this was confirmed in WHO susceptibility tests against both M- and S-form *An. gambiae s.s*. (mortality 100% 24 h after 1 hour exposure, N = 50 for M-form, N = 25 for S-form).

A total of 30 isofemale *An. gambiae s.s*. families from Soumousso (14 S-form, 3 M-form and 13 *An. arabiensis*) were exposed to bendiocarb sprayed walls at three to five days and 17-19 days old. The total number of mosquitoes exposed to each wall and the number dead per isofemale line are presented in Table 4. In order to estimate whether mosquito age had an effect on susceptibility, the exposure time was reduced to 3 minutes. There was no observed difference in mortality between any of the sprayed walls (Additional file 1) and as a result, 'Treatment' was dropped from the model. The final fitted model included 'Age' and 'Species' as fixed variables and the estimated parameters, z-values and p-values are presented in the Additional Information (Additional file 1).

**Table 4:** Mortality within *Anopheles gambiae s.l*. isofemale lines exposed to bendiocarb-sprayed walls

			three to five days			17-19 days		
**WALL**	**Family No**.	**Species**	**N**	**No. Dead**	**% Mortality**	**N**	**No. Dead**	**% Mortality**

MUD#1	313	M	11	2	18.2	10	5	50.0

	413	*An. arabiensis*	9	0	0.0	11	5	45.5

	423	*An. arabiensis*	10	1	10.0	12	2	16.7

	431	*An. arabiensis*	11	2	18.2	10	8	80.0

	432	*An. arabiensis*	11	2	18.2	11	6	54.5

	441	*An. arabiensis*	11	2	18.2	10	6	60.0

	465	*An. arabiensis*	12	0	0.0	10	8	80.0

	523	S	11	4	36.4	8	3	37.5

	528	S	13	1	7.7	8	5	62.5

	544	S	12	0	0.0	8	4	50.0

CEMENT#1	499	S	10	3	30.0	8	4	50.0

	537	*An. arabiensis*	11	2	18.2	9	5	55.6

	557	S	11	2	18.2	11	6	54.5

	568	*An. arabiensis*	12	2	16.7	10	5	50.0

	619	S	11	3	27.3	10	2	20.0

	631	*An. arabiensis*	9	2	22.2	10	7	70.0

	637	*An. arabiensis*	12	2	16.7	9	5	55.6

	663	*An. arabiensis*	12	0	0.0	7	1	14.3

	685	S	12	3	25.0	10	5	50.0

	782	S	10	1	10.0	10	5	50.0

MUD#2	504	*An. arabiensis*	12	1	8.3	13	0	0.0

	516	*An. arabiensis*	12	3	25.0	9	0	0.0

	536	S	12	5	41.7	10	3	30.0

	551	S	10	1	10.0	12	7	58.3

	566	S	11	5	45.5	9	2	22.2

	569	M	12	1	8.3	9	0	0.0

	609	M	9	2	22.2	10	5	50.0

	680	S	10	2	20.0	11	7	63.6

	754	S	13	5	38.5	10	5	50.0

	768	S	12	2	16.7	10	4	40.0

*M = *An. gambiae s.s*. M-form; S = *An. gambiae s.s*. S-form

There was strong evidence that the age had an effect on mortality and older mosquitoes (17-19 days) were more likely to be killed on exposure to the sprayed-walls used in this experiment (OR = 3.39, CI 95% = 2.35-4.90). There was little evidence that the species status of the isofemale line had any effect on mortality rates (Table 3).

## Discussion

Two findings with important implications for malaria control emerge from this study, one discouraging and the second potentially more positive. Firstly, LLINs selected for use in this study are not adequately killing malaria vectors. Secondly, malaria vectors become increasingly susceptible to insecticide exposure as they age.

The poor performance of LLINs in the field highlights the importance of follow-up monitoring and evaluation after net distribution, particularly in areas where resistance may be further eroding net efficacy. This finding is, admittedly, based on a small sample size of three nets but these were selected at random from LLINs distributed by the NMCP in the past two years, i.e. well within the intended lifespan of the nets. No information on the number of washes is available but the insecticide concentration has decreased to equal or less than 4% of that of a new Permanet 2.0^® ^and the mortality in the susceptible laboratory population is very low (< 53%) for two of the nets. For field populations from Burkina Faso, where resistance is prevalent, the mortality from the nets was disappointing when tested according to standard WHO procedures for assessing LLIN performance [30] (Figure 3).

Fortunately, as part of the NMCP of Burkina Faso, there are immediate plans to increase the number of houses covered with IRS to supplement the recent distribution of LLINs across the country. More than 118,000 people in the Diebougou district of south-west Burkina Faso have been protected through a recent IRS campaign [4]. Although the carbamate bendiocarb offers a promising alternative to pyrethroids, resistance has emerged in pockets of West Africa associated with the *ace-1 *mutation. In Burkina Faso, *ace-1 *is prevalent at moderate frequencies in *An. gambiae s.s*. from the south-west [14] and the introduction of bendiocarb for IRS in this region would represent an additional selection pressure on this mutation.

The second more positive finding is the main focus of this study. The results presented here confirm the findings from previous studies, which show a reduction in phenotypic insecticide resistance in older mosquitoes [22-27]. The F1 progeny of *An. gambiae s.s*. S-forms collected from Soumousso were fully susceptible to deltamethrin (0.05%) at 17-19 days old, despite being fully resistant at a younger age, according to WHO criteria (Figure 1). Earlier studies on this subject have focussed largely on laboratory colonies or have only been able to analyse a mixture of sub-species from the wild. The present study demonstrates that highly resistant insects from the field lose tolerance to insecticides at an older age.

When evaluated against either LLINs or sprayed walls in Burkina Faso, the potential operational impact of age-dependent decreases in phenotypic resistance becomes evident. Nets that failed to kill young mosquitoes were still partially protective against older mosquitoes. Similarly, while bendiocarb IRS is currently effectively killing both age cohorts after a 30-min exposure, an age dependent effect is clearly visible after shorter exposures (Table 3).

A decrease in insecticide resistance with mosquito age may have significant epidemiological consequences for vector control in areas of wide LLIN and IRS coverage. The development of malaria parasites into the infectious sporozoite stage takes 10 or more days following a blood-meal [35]. The older cohort of *An. gambiae s.s*. used in this study (17-19 days old) could potentially harbour infectious parasites but would be more susceptible to insecticide-based control. Targeting older, and arguably more epidemiologically significant mosquitoes, has been proposed as an alternative strategy for wider malaria control in which the selection on resistance to the control agent is reduced [36]. Natural senescence processes may contribute to the vulnerability of the older population in contact with any insecticide-based intervention. The importance of younger mosquitoes on the vectorial capacity should not, however, be overlooked, as the age at which a mosquito bites an infectious host is a key determinant on the potential to transmit the parasite to additional hosts [37]. The development of more advanced age-grading technologies offers the opportunity for further exploration of insecticide resistance in the context of the age-structure of local *Anopheles *vector populations [38].

It is widely assumed that mosquitoes possessing insecticide resistance traits will survive repeated exposures on encountering a LLIN or a sprayed house. Under this scenario, insecticide-based control efforts will fall short in reducing the mean population and more importantly, the mean survival rate of the vector, and as a result, fail to reduce the vectorial capacity below the threshold for disease control. There are, however, very few examples where a link between the presence of insecticide resistance and vector control failure has been demonstrated. If insecticide resistance and the associated mechanisms are so widespread amongst malarial vectors, why are vector control failures so few and far between? Part of the answer is undoubtedly that the appropriate experiments have not yet been performed to investigate the epidemiological impact of resistance. Studies extending over several transmission seasons that definitively demonstrate reduced survival of mosquitoes against insecticide-treated surfaces are very rare in the literature and this knowledge gap urgently needs to be filled.

## Competing interests

This work was partially funded by BayerCropScience and they provided the bendiocarb for the IRS. BayerCropScience played no role in the experimental design or interpretation of the results.

## Authors' contributions

HR and SN conceived the study. All authors participated in the study design. CJ and AS oversaw field collections, bioassays and conducted laboratory work. CJ wrote a draft of the manuscript. CJ and PCDJ performed the statistical analysis. All authors read and approved the final manuscript.

## Supplementary Material

Additional File 1 **Details the rationale and model parameters for the LLIN and IRS experiments and includes the following tables and figures**. **Table A1 **The Akaike's Information Criteria (AIC) for model selection for the *An. gambiae s.s*. isofemale LLIN and IRS experiments. **Table A2 **The Akaike's Information Criteria (AIC) for model selection for the *An. gambiae s.s*. isofemale LLIN experiment excluding data for *An. arabiensis*. **Table A3 **Estimates of parameters, standard errors, 95% confidence intervals, *z *and *p *values for mortality in response to LLINs. **Table A4 **Estimates of parameters, standard errors, 95% confidence intervals, *z *and *p *values for mortality in response to bendiocarb-sprayed walls. **Table A5 **Individual estimates of parameters from re-fitted model with Age:Treatment interaction term for each treatment reference category. **Figure A1 **Mortality of isofemale lines in response to bendiocarb treated walls pooled by age. Click here for file
