# Supplementary material for: Aging partially restores the efficacy of malaria vector control in insecticide-resistant populations of Anopheles gambiae s.l. from Burkina Faso
Source: Malar J. 2012 Jan 23;11:24. doi: 10.1186/1475-2875-11-24 (PMC3312828; doi:10.1186/1475-2875-11-24)
Supplement: Additional File 1 — Details the rationale and model parameters for the LLIN and IRS experiments and includes the following tables and figures. Table A1 The Akaike's Information Criteria (AIC) for model selection for the An. gambiae s.s. isofemale LLIN and IRS experiments. Table A2 The Akaike's Information Criteria (AIC) for model selection for the An. gambiae s.s. isofemale LLIN experiment excluding data for An. arabiensis. Table A3 Estimates of parameters, standard errors, 95% confidence intervals, z and p values for mortality in response to LLINs. Table A4 Estimates of parameters, standard errors, 95% confidence intervals, z and p values for mortality in response to bendiocarb-sprayed walls. Table A5 Individual estimates of parameters from re-fitted model with Age:Treatment interaction term for each treatment reference category. Figure A1 Mortality of isofemale lines in response to bendiocarb treated walls pooled by age. [file 1475-2875-11-24-S1.DOC]

**Aging partially restores the efficacy of malaria vector control in insecticide resistant populations of *Anopheles gambiae* s.l from Burkina Faso**

**ADDITIONAL FILE 1**

Christopher M. Jones^1^, Antoine Sanou^2^, Wamdaogo M. Guelbeogo^2^, Sagnon N’Fale^2^ and Hilary Ranson^1^

^1^Liverpool School of Tropical Medicine, Pembroke Place, Liverpool, L3 5QA

^2^Centre National de Recherche et de la Formation sur Paludisme, 01 BP2208, Ouagadougou, Burkina Faso

*Corresponding author, Email: jonescm@liverpool.ac.uk

## Statistical analysis for long-lasting insecticide net and bendiocarb IRS assays

Mosquito mortality was measured in two age cohorts (3-5 days and 17-19 days) exposed to field-based LLINs and IRS to estimate whether age has an effect on being killed by the insecticide.

The response to each treatment (net or wall) was treated as the proportion dead or alive and considered as a binary response variable. A separate model was inferred for the two experiments and data fitted using a binomial Generalised Linear Mixed Model (GLMM) with a logit link function. Mortality was modelled as a function of three fixed factors termed ‘Age’ (A = younger (3-5days) or B = older (17-19 days)); ‘Species’ (A = *An. arabiensis*, M = *An. gambiae s.s.* M-form or S = *An. gambiae s.s.* S-form); ‘Treatment’ (T1-T3, the net or wall used). The term ‘Family’ was fitted as a random effect to allow the log odds of mortality to vary between families. The models were fitted *a priori*.

The estimated parameters, standard errors, confidence limits, *z* values and *p* values for the final fitted models presented in the main text are given in Tables A2 and A3. The fit of each model was validated by plotting the residuals against the fitted model values and each term included in the model. The estimated odds ratio with 95% confidence intervals was calculated from the exponential of the intercept and estimated parameters for each treatment relevant to the control.

*Justification for GLMM approach for LLIN assays*

Logistic regression models which test for interactions between two explanatory variables encounter problems in the presence of zero cell counts [33]. This can result in infinite estimates for the model parameters. In this study, there was a zero cell count (zero mortality) in the younger age-group exposed to LLIN#1. To overcome this problem, and to test for evidence of an interaction, a small constant (0.5) was added to the mortality rate of those rows contributing to the zero cell count. Because this approach conservatively inflates mortality in the reference group LLIN#1, the model was re-fitted for each ‘Age’ and ‘Treatment’ reference group, to give individual mortality estimates (A5).

Another issue with our approach was the imbalance between replicates for species and treatment (e.g. no *An. arabiensis* families were tested against LLIN#3). Although there was no *a priori* belief that any observed effect on mortality by age would be influenced by species, a separate analysis was conducted whereby *An. arabiensis* were removed from the data set to test for an interaction between species and the two other parameters (‘Age’ and ‘Treatment’). The models were selected and tested according to the criteria outlined above and the results presented in Table A2. The model which best fit the data included all factors plus the interaction between ‘Age’ and ‘Treatment’ followed by the model including just all terms (ΔAIC = 11.7). According to both AIC and likelihood ratio tests (LRT), there was no evidence to suggest an interaction between species (*An. gambiae s.s.* S- and M-forms) and treatment or age (Likelihood Ratio Test = *p* > 0.05).

*Justification for GLMM approach for IRS assays*

No differences in mortality were observed between isofamily lines in response to bendiocarb-sprayed walls (Figure A1). On this basis, ‘Treatment’ was subsequently dropped from the IRS model and justified according to the selection criteria (ΔAIC = 2.76) (Table A1).

**TABLES**

**TABLE A1.** The Akaike’s Information Criteria (AIC) for model selection for the *An. gambiae s.l.* isofemale LLIN and IRS experiments.

| **Experiment** | **Model** | **AIC** | **ΔAIC** |
| --- | --- | --- | --- |
| *LLIN^a^* | ‘Age'+'Species'+'Treatment'+’Age:Treatment’+(1|'Family') | 97.5 | - |
|  | ‘Age'+'Species'+'Treatment'+(1|'Family') | 110.7 | 13.2 |
|  | ‘Age'+'Species'+(1|'Family') | 115.3 | 17.8 |
|  | ‘Age'+'Treatment'+(1|'Family') | 120.7 | 23.2 |
|  |  |  |  |
| *IRS* | ‘Age'+'Species'+(1|'Family') | 105.6 | - |
|  | ‘Age'+'Species'+'Treatment'+(1|'Family') | 108.3 | 2.7 |
|  | ‘Age'+'Treatment'+(1|'Family') | 108.7 | 3.1 |
|  |  |  |  |

^a^A small constant was added to rows contributing to zero cell counts and the GLMM is modelled on this data for the LLIN experiment.

**TABLE A2** The Akaike’s Information Criteria (AIC) for model selection for the *An. gambiae s.l.* isofemale LLIN experiment excluding data for *An. arabiensis*

| **Model** | **AIC** | **ΔAIC** |
| --- | --- | --- |
| ‘Age'+'Species'+'Treatment'+'Age:Treatment'+(1|'Family') | 73.2 | - |
| ‘Age'+'Species'+'Treatment'+(1|'Family') | 84.9 | 11.7 |
| ‘Age'+'Species'+'Treatment'+'Age:Species'+(1|'Family') | 85.4 | 12.2 |
| ‘Age'+'Species'+'Treatment'+'Treatment:Species'+(1|'Family') | 87.2 | 14.0 |

**TABLE A3.** Estimates of parameters, standard errors, 95% confidence intervals, *z* and *p* values for mortality in response to LLINs.

|  |  | **Estimate** | **SE** | **Lower 95% CI** | **Upper 95% CI** | ***z* value** | ***p* value** |
| --- | --- | --- | --- | --- | --- | --- | --- |
| LLIN |  |  |  |  |  |  |  |
|  |  |  |  |  |  |  |  |
|  | Intercept | -1.4585 | 0.6039 | -2.642 | -0.275 | -2.415 | 0.0157 |
|  |  |  |  |  |  |  |  |
| Age |  |  |  |  |  |  |  |
|  | Young (3-5d) | - | - | - | - | - | - |
|  | Old (17-19d) | 1.7050 | 0.3261 | 1.066 | 2.344 | 5.228 | 1.71E-07 |
|  |  |  |  |  |  |  |  |
| Species |  |  |  |  |  |  |  |
|  | *An. arabiensis* | - | - | - | - | - | - |
|  | *An. gambiae* S-form | -3.2222 | 0.7971 | -4.784 | -1.660 | -4.042 | 5.29E-05 |
|  | *An. gambiae* M-form | -2.0446 | 0.6061 | -3.232 | -0.857 | -3.373 | 0.0007 |
|  |  |  |  |  |  |  |  |
| Treatment |  |  |  |  |  |  |  |
|  | LLIN#1 | - | - | - | - | - | - |
|  | LLIN#2 | 1.7549 | 0.5484 | 1.116 | 2.394 | 3.200 | 0.0014 |
|  | LLIN#3 | 0.0551 | 0.7664 | -0.584 | 0.694 | 0.072 | 0.9427 |

**TABLE A4.** Estimates of parameters, standard errors, 95% confidence intervals, *z* and *p* values for mortality in response to bendiocarb-sprayed walls.

|  |  | **Estimate** | **SE** | **Lower 95% CI** | **Upper 95% CI** | ***z* value** | ***p* value** |
| --- | --- | --- | --- | --- | --- | --- | --- |
| IRS |  |  |  |  |  |  |  |
|  |  |  |  |  |  |  |  |
|  | Intercept | -1.7557 | 0.2125 | -2.172 | -1.339 | -8.263 | < 2e-16 |
|  |  |  |  |  |  |  |  |
| Age |  |  |  |  |  |  |  |
|  | Young (3-5d) | - | - | - | - | - | - |
|  | Old (17-19d) | 1.2216 | 0.1874 | 0.854 | 1.589 | 6.519 | 7.09E-11 |
|  |  |  |  |  |  |  |  |
| Species |  |  |  |  |  |  |  |
|  | *An. arabiensis* | - | - | - | - | - | - |
|  | *An. gambiae* S-form | 0.4456 | 0.2415 | -0.028 | 0.919 | 1.845 | 0.0650 |
|  | *An. gambiae* M-form | -0.0763 | 0.4182 | -0.896 | 0.743 | -0.182 | 0.8550 |

**TABLE A5. Individual estimates of parameters from re-fitted model with Age:Treatment interaction term for each Treatment reference category.**

| **Ref Level** | **Model Parameter*** | **Estimate** | **SE** | ***z* value** | ***p* value** |
| --- | --- | --- | --- | --- | --- |
| Age = Young (3-5d); Treatment = LLIN#1 | Intercept | -4.0986 | 1.5761 | -2.601 | 0.00931 ** |
|  | **Old (17-19d)** | **4.6647** | **1.522** | **3.064** | **0.00218 **** |
|  | *An. gambiae* S-form | -2.0142 | 0.575 | -4.17 | 3.04e-05 *** |
|  | *An. gambiae* M-form | -3.1494 | 0.7552 | -3.5030 | 0.00046 *** |
|  | LLIN#2 | 4.8514 | 1.5908 | 3.05 | 0.00229 ** |
|  | LLIN#3 | 3.4740 | 1.7307 | 2.007 | 0.04472 * |
|  | Old (17-19d):LLIN#2 | -3.8252 | 1.5784 | -2.423 | 0.01538 * |
|  | Old (17-19d):LLIN#3 | -4.1469 | 1.7355 | -2.3900 | 0.01687 * |
| Age = Young (3-5d); Treatment = LLIN#2 | Intercept | 0.7528 | 0.5322 | 1.414 | 0.15724 |
|  | **Old (17-19d)** | **0.8395** | **0.422** | **1.99** | **0.04659 *** |
|  | *An. gambiae* S-form | -3.1494 | 0.7552 | -4.17 | 3.04e-05 *** |
|  | *An. gambiae* M-form | -2.0142 | 0.575 | -3.503 | 0.00046 *** |
|  | LLIN#1 | -4.8514 | 1.5908 | -3.05 | 0.00229 ** |
|  | LLIN#3 | -1.3774 | 0.9511 | -1.448 | 0.14757 |
|  | Old (17-19d):LLIN#1 | 3.8252 | 1.5784 | 2.4230 | 0.01538 * |
|  | Old (17-19d):LLIN#3 | -0.3217 | 0.9343 | -0.344 | 0.7306 |
| Age = Young (3-5d); Treatment = LLIN#3 | Intercept | -0.6246 | 0.9746 | -0.641 | 0.5216 |
|  | **Old (17-19d)** | **0.5178** | **0.834** | **0.621** | **0.53444** |
|  | *An. gambiae* S-form | -2.0142 | 0.575 | -4.17 | 3.04e-05 *** |
|  | *An. gambiae* M-form | -3.1494 | 0.7552 | -3.5030 | 0.00046 *** |
|  | LLIN#1 | -3.4740 | 1.7307 | -2.007 | 0.04472 * |
|  | LLIN#2 | 1.3774 | 0.9511 | 1.448 | 0.14757 |
|  | Old (17-19d):LLIN#1 | 4.1468 | 1.7355 | 2.3890 | 0.01687 * |
|  | Old (17-19d):LLIN#2 | 0.3217 | 0.9343 | 0.344 | 0.7306 |

*Estimates of mortality (log odds) for the older age group (17-19d) are highlighted in bold.

**FIGURE LEGENDS**

**Figure A1.** A boxplot representing the percentage mortality of *An. gambiae* s.l. isofemales from Soumousso exposed to bendiocarb-sprayed walls.

The mortality within isofemales lines is pooled for each age cohort to demonstrate the uniformity of mortality between wall treatments. Mosquitoes were exposed to bendiocarb for 3 minutes rather than the WHO standard protocol of 30 minutes to show an age-effect. The number of isofamilies exposed to each wall is given above the boxplots.

**Figure A1.**
